# Highly Conductive PEDOT:PSS Thin Films with Two-Dimensional Lamellar Stacked Multi-Layers

**DOI:** 10.3390/nano10112211

**Published:** 2020-11-06

**Authors:** Youngno Kim, Yunryeol Kim, Jung Hyun Kim

**Affiliations:** Department of Chemical and Biomolecular Engineering, Yonsei University, 134 Shinchon-Dong, Seodaemoon-Gu, Seoul 03722, Korea; dudsh3@yonsei.ac.kr (Y.K.); ryeol226@yonsei.ac.kr (Y.K.)

**Keywords:** conjugated polymers, PEDOT:PSS, organic electrode, carrier mobility, electrical conductivity

## Abstract

Conjugated polymers are desired as organic electrode materials because of their functional properties such as solution process, low cost, and transparency. Poly(3,4-ethylenedioxythiophene):poly(styrenesulfonate) (PEDOT:PSS), in particular, shows the highest applicability, but its heterogeneous structure presents limitations in terms of electrical conductivity. In this study, a facile method to fabricate multi-layered thin films with higher ordered structures was developed. Through the etching process with H_2_SO_4_ and dimethyl sulfoxide(DMSO), the insulated rich-PSS was removed from the upper layer to improve its electrical properties and rearrange the PEDOT molecular structures. The thickness of PEDOT:PSS thin films was experimentally optimized to maximize the enhancement of carrier mobility via a layer-by-layer (LBL) process. The combined method, consisted of etching and the LBL process, showed the improvement of the charge carrier mobility from 0.62 to 2.80 cm^2^ V^−1^ s^−1^. The morphology and crystallinity of the ordered PEDOT:PSS structure were investigated by X-ray photoemission spectroscopy (XPS), Raman, and X-ray diffraction (XRD). As a result, two-dimensional lamellar-stacked PEDOT:PSS thin films were fabricated through the repetitive etching and LBL process. The optimized PEDOT:PSS thin film showed an excellent electrical conductivity of 3026 S cm^−1^, which is 3.8 times higher than that of the pristine film (801 S cm^−1^).

## 1. Introduction

The need for electronic devices in everyday life increases daily. Indium tin oxide (ITO) is used as the electrode for most electronic devices requiring transparency. However, ITO can be depleted, as it is a rare metal, and it has to be processed through a difficult method, called the deposition process, under high-temperature conditions. Most inorganic materials have the disadvantage of being fragile. Recently, flexible, bendable, and stretchable devices have been in the limelight in the electronic industry [1,2,3]. For these reasons, organic electrode-based conjugated polymers have received attention for their application as electrodes in wearable devices, such as a stretchable sensors [4]. Conducting polymers based on a conjugation structure have electrical activity through a π–π interaction overlap by the sp^2^ hybrid orbital [5]. The most desired conjugated polymer, poly(3,4-ethylenedioxythiophene):poly(styrenesulfonate) (PEDOT:PSS), has many advantages such as solution-based processes, transparency in the visible light range, and low-temperature processing. The PSS of PEDOT:PSS acts as a stabilizer in water-based dispersion and an acid dopant for PEDOT grains with coulombic interactions [6]. However, limitations of their electrical conductivity with respect to inorganic materials remain. To overcome this disadvantage, many studies have focused on the removal of insulated PSS through a dipping process using solvents such as ethylene glycol(EG) and DMSO, and acid treatment [7,8]. In addition, some studies have focused on the enhancement of the electrical conductivity of PEDOT:PSS through layer-by-layer structures [9,10]. The two precedent methods have different mechanisms for improving electrical conductivity. The first etching process using an organic solvent physically removed the insulated upper layer consisting of rich-PSS. The second layer-by-layer process formed repetitive two-dimensional structures and made it possible to transfer the hole easily. 

In this work, the advantages of the two precedent processes converged to improve the electrical conductivity of PEDOT:PSS thin films. In particular, the electrical properties of PEDOT:PSS were greatly influenced by the coating and baking conditions [11]. The detailed parameters, such as film thickness and annealing temperature, were optimized in prior studies [12]. Additionally, PEDOT:PSS has intrinsically low carrier mobility compared to inorganic materials because of its amorphous structure [13]. The H-PEDOT:PSS thin film treated by the H_2_SO_4_/DMSO post-treatment and layer-by-layer (LBL) process showed the improved carrier mobility, which resulted in the enhancement of electrical conductivity. As the insulated rich-PSS layers were removed, the ordered structure was formed with rich-PEDOT, which is electrically conductive, and a synergistic effect was confirmed through the combined process. As a result, the optimized process consisted of repetitive spin-coating and etching, forming two-dimensional lamellar stacked multi-layers. The fabricated H-PEDOT:PSS thin film was analyzed by X-ray photoemission spectroscopy (XPS), atomic force microscopy (AFM), X-ray diffraction (XRD) and Raman.

## 2. Materials and Methods 

### 2.1. Materials

3,4-Ethylenedioxythiophene (EDOT; 97%), poly(4-styrene sulfonate acid) (PSSA), iron(III) sulfate (Fe_2_(SO_4_)_3_, 97%), sodium persulfate (Na_2_S_2_O_8_, ≥99.0%), sulfuric acid (H_2_SO_4_, 95%), dimethyl sulfoxide (DMSO), and isopropyl alcohol (IPA) were purchased from Sigma-Aldrich (Yongin, Korea). The ion exchange resin (cation and anion) was purchased from Samyang Co. (Seoul, Korea). 

### 2.2. Fabrication of PEDOT:PSS Thin Films

The synthesis of PEDOT:PSS followed the Baytron P procedure, and the ratio of PEDOT:PSS was 1:2.5 wt% [14]. Remaining impurities such as sodium ions were removed by ion exchange for 1 h 30 min at room temperature. Then, 5 wt% DMSO and 0.1 wt% wetting agent were added to the PEDOT:PSS dispersions and stirred for 10 min. The solution was dropped onto a bare glass substrate (75 × 75 mm^2^) and coated via spin-coating for 30 s. The film with the applied solution was dried at 150 °C in a convection oven for 10 min. The thickness of the PEDOT:PSS thin film was experimentally set by optimized spin-coating conditions to achieve a uniform thickness of about 60 nm after H_2_SO_4_/DMSO post-treatment. 

### 2.3. Fabrication of Two-Dimensional Lamellar Stacked PEDOT:PSS Thin Films

**Scheme 1:**
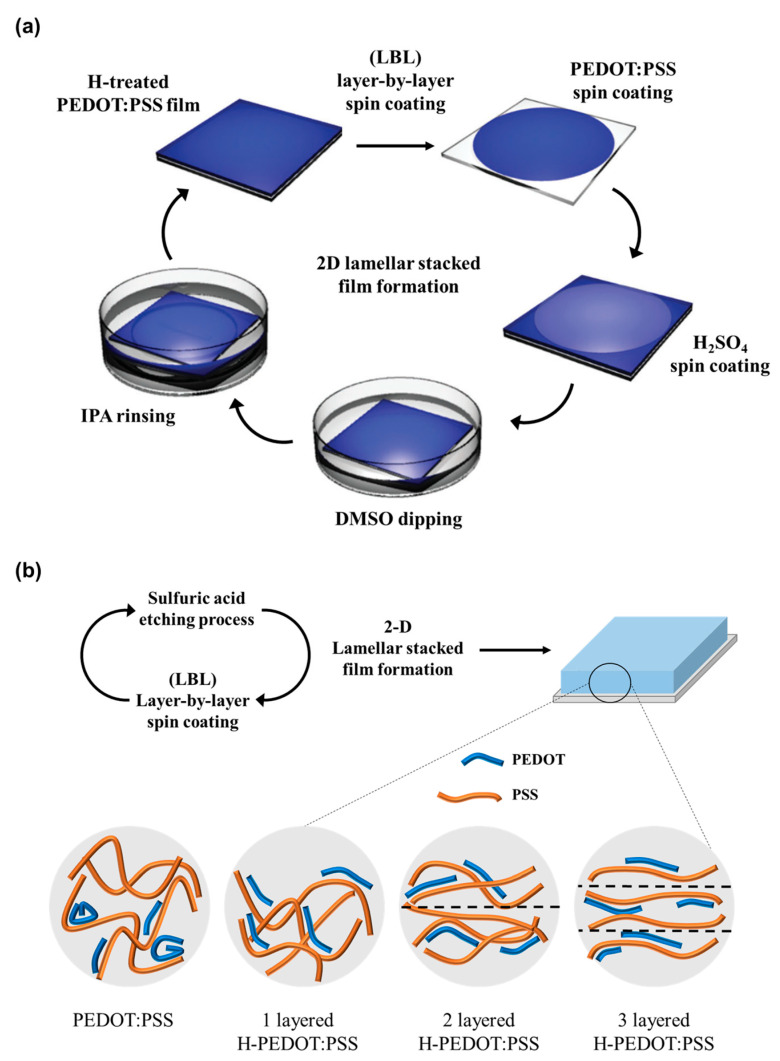
(**a**) Schematic illustration of H_2_SO_4_/DMSO post-treatment of the PEDOT:PSS film and (**b**) Lamellar stacked structure of H-Poly(3,4-ethylenedioxythiophene):poly(styrenesulfonate) (PEDOT:PSS) film through H_2_SO_4_/DMSO post-treatment and layer-by-layer (LBL) method.

A H_2_SO_4_/DMSO-treated PEDOT:PSS film was fabricated by removing the upper layer of the rich-PSS using the etching process as reported in our previous work [12]. The optimized 15-M H_2_SO_4_ was dropped on the PEDOT:PSS thin film, which was entirely covered and blown away by 1000 rpm for 30 s. The PEDOT:PSS thin film was annealed at 150 °C for 5 min and immersed in DMSO solution for 2 min. The H_2_SO_4_/DMSO-treated PEDOT:PSS thin film was rinsed in an IPA solution for 2 min and finally dried at 120 °C for 5 min in a convection oven. On the H_2_SO_4_/DMSO-treated PEDOT:PSS film, the layer-by-layer (LBL) spin-coating and H_2_SO_4_/DMSO post-treatment processes were repeated to form a two-dimensional lamellar stacked structure (Scheme 1). The final thicknesses of the PEDOT:PSS thin films that consisted of single, double, and multi-layers were optimized at 60 nm to clarify the effect of the number of layers. For structures with more than three layers, there were no dramatic improvements in characteristics and the deviation of the measured values was large, so they were excluded from the data.

### 2.4. Characterization

To confirm the removal of PSS and the ratio of PEDOT:PSS through etching post-treatment, X-ray photoemission spectroscopy (XPS; Thermo Scientific K-alpha, East Grinstead, UK) was performed. The morphologies of the PEDOT:PSS films were examined using atomic force microscopy (AFM; XE-100, Park Systems Co., Suwon, Korea) with topographic and phase images. High-resolution X-ray diffraction (HR-XRD; SmartLAB, Rigaku Co., Tokyo, Japan) and Raman spectroscopy (LabRAM Aramis, Horiba Jobin Yvon, NJ, USA) using a 633-nm He–Ne laser as the excitation source were used to examine the lamellar two-dimensional structures of PEDOT:PSS films. To measure the sheet resistance of PEDOT:PSS films, a 4-point probe resistivity system (RT-70V, Napson Co., Chiba, Japan) was used. Then, the film thickness was measured using a Surface Profiler (DektakXT stylus profiler, Bruker, Milan, Italy) and added to the formula to obtain the electrical conductivity. Finally, the carrier mobility of the PEDOT:PSS thin film was directly measured by Hall effect measurements (HMS-3000) at room temperature.

## 3. Results and Discussion

PEDOT:PSS consists of hydrophobic PEDOT and hydrophilic PSS, which can make PEDOT disperse in water. They are connected with ionic interactions, and PSS functions as both an acid dopant and a stabilizer in water dispersion. However, PSS is treated as an insulating impurity after the film formation process [15]. In particular, free-PSS that is not complexed with PEDOT has amorphous regions and disturbs the hopping mechanism between the PEDOT grains. To induce the inherent properties of PEDOT, primarily organic solvents such as ethylene glycol, dimethyl sulfoxide, or dimethylformamide were added to the solution for phase separation between PEDOT and PSS. However, after separation, as the PEDOT:PSS dispersion was baked at a high temperature, the rich-PEDOT layer formed toward the substrate and the rich-PSS was located at the top of the film [16]. The etching post-treatment for the removal of the upper rich-PSS layer, which is covered on the rich-PEDOT layer, was studied in our previous work [12].

**Figure 1:**
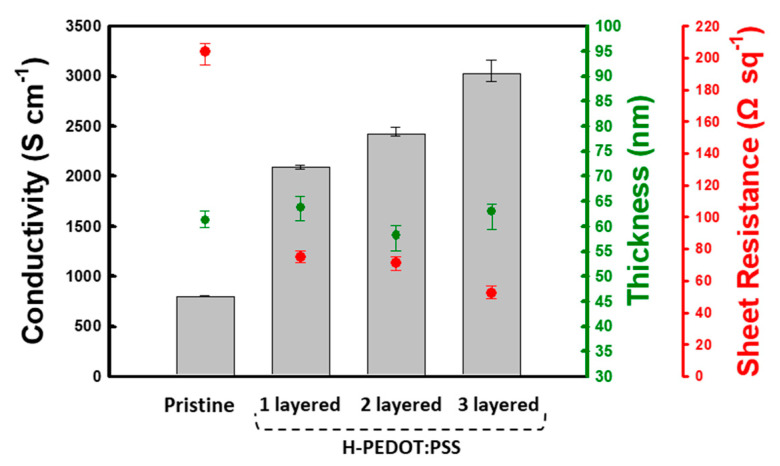
The comparison data of sheet resistance (red color), film thickness (green color) and electrical conductivity of pristine PEDOT:PSS and H-PEDOT:PSS thin films according to an H_2_SO_4_/DMSO treatment and an layer-by-layer (LBL) process. The thickness of each sample was experimentally optimized at the same thickness (about 60 nm).

In this work, an additional improvement of electrical conductivity was identified through a combination of a previously developed etching post-treatment and a simple LBL process. The structure of the PEDOT:PSS thin film changed from heterogeneous to two-dimensional lamellar stacked and ordered structures by the combination of the H_2_SO_4_/DMSO post-treatment and the LBL process. The ordered PEDOT:PSS thin film, fabricated by the combined process, was termed as H-PEDOT:PSS. As shown in Figure 1, the sheet resistance, film thickness, and electrical conductivity of the PEDOT:PSS and H-PEDOT:PSS thin films were measured. The term “H” means the H_2_SO_4_/DMSO post-treatment and “S” means spin-coating by an LBL process. Based on this facile method, H-PEDOT:PSS thin films consisting of single, double, and multi-layer films were fabricated, and the final thickness of each sample was adjusted to 60 nm. The electrical conductivities (σ) of the H-PEDOT:PSS thin films were evaluated using the following equation:

(1) $$\sigma \;\left({\mathrm{cm}}^{-1}\right)=\frac{1}{\mathrm{sheet}\;\mathrm{resistance}\;\times \mathrm{film}\;\mathrm{thickness}}$$

The pristine PEDOT:PSS thin film without post-treatment showed relatively high sheet resistance, 204 Ω sq^−1^, while the sheet resistance of one-layered H-PEDOT:PSS was 75 Ω sq^−1^. Through H_2_SO_4_/DMSO post-treatment, the thickness of the PEDOT:PSS thin film was reduced from 170 to 64 nm (62%) and the initial value of sheet resistance 75 Ω sq^−1^ was maintained, as shown in Table 1. Since the thickness of both samples is about 60 nm, the difference in sheet resistance can be explained by the presence of PSS, which constitutes the film thickness. The film thickness of the one-layered H-PEDOT:PSS decreased while the sheet resistance was simultaneously maintained, so the electrical conductivity increased rapidly to 2091 S cm^−1^. Compared to one-layered H–PEDOT:PSS, two- and three-layered H–PEDOT:PSS showed lower sheet resistances at the same thickness. This tendency indicates that the repetitive H_2_SO_4_/DMSO post-treatment and LBL process make the PEDOT chains dense and ordered. As a result, the electrical conductivity was gradually increased from 2091 (one-layered H-PEDOT:PSS) to 3026 S cm^−1^ (three-layered H–PEDOT:PSS) because of the two-dimensional lamellar stacked structures. In previous studies, there were similar concepts of improvement for electrical properties through layer-by-layer processes [17]. Sun et al. reported a polar solvent-aided LBL technique to fabricate nanocomposite films comprising PEDOT:PSS and Sb_2_Te_3_ and the three-layered sample was optimized. Likewise, the electrical conductivity was dramatically enhanced by H_2_SO_4_/DMSO post-treatment and LBL process and saturated at the three-layered H-PEDOT:PSS thin film in this work.

**Figure 2:**
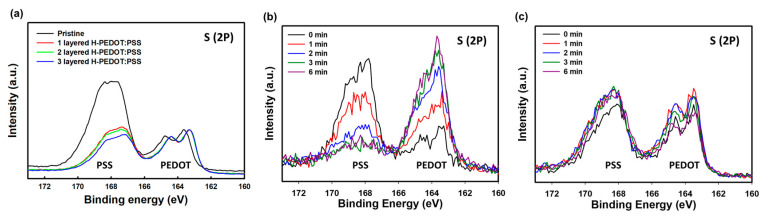
(**a**) XPS of the pristine PEDOT:PSS film and PEDOT:PSS films with sulfuric acid etching treatment and XPS depth profile etching by argon ion of (**b**) the pristine PEDOT:PSS thin film and (**c**) three-layered H-PEDOT:PSS thin film.

Figure 2a shows that the free-PSS was completely removed from the H-PEDOT:PSS thin films. When the PEDOT peak (163–165 eV) was normalized, the PSS peak (167–169 eV) was decreased by H_2_SO_4_/DMSO post-treatment [18,19]. In addition, depending on the repetitive process, the PSS peak was slightly reduced, except for the minimum amount combined with the PEDOT chain. The ratio between PEDOT and PSS of pristine PEDOT:PSS and three-layered H-PEDOT:PSS was analyzed by the XPS depth profile (Figure 2b,c). From the top surface of the pristine PEDOT:PSS thin film to bottom substrate, the concentration of PSS gradually decreased as the argon ion cluster etched at pristine sample. However, the three-layered H-PEDOT:PSS thin film showed a completely consistent ratio between PSS and PEDOT as a function of depth of the film, which means that the free-PSS was neatly removed by the H_2_SO_4_/DMSO treatment and LBL process.

**Figure 3:**
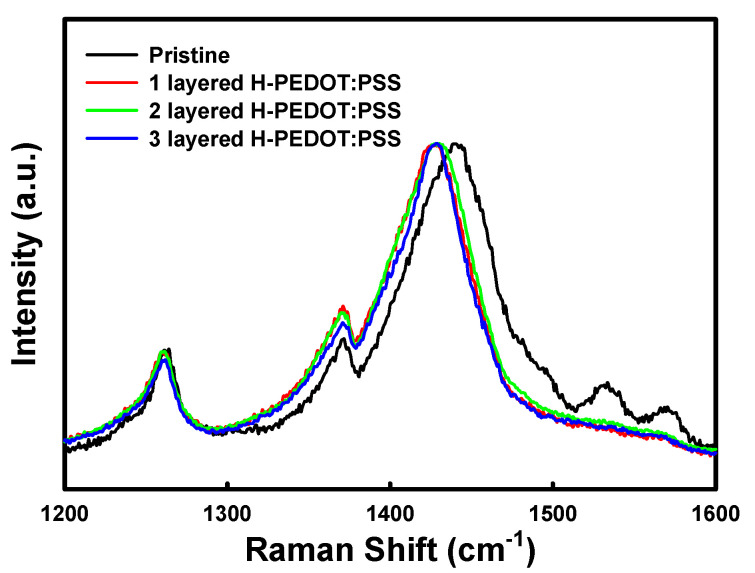
Raman spectra of the pristine PEDOT:PSS film and PEDOT:PSS films with sulfuric acid etching treatment (under 632.8 nm He–Ne laser excitation).

To observe the conformational change of the PEDOT:PSS thin films, the Raman spectra were measured. The Raman band between 1200 and 1600 cm^−1^ corresponds to the stretching vibration C_α_ = C_β_ bonds of PEDOT chains, as shown in Figure 3. The benzoid vibration peak (1450 nm^−1^) shifted to the quinoid vibration peak (1415 nm^−1^) through the H_2_SO_4_/DMSO treatment [20]. This showed the change of PEDOT chains from coiled to aligned structures. Furthermore, the two peaks at 1535 and 1570 cm^−1^ indicate that the PSS peak was completely reduced by the H_2_SO_4_/DMSO post-treatment. There was little difference in the shift of the peak with the repetitive H_2_SO_4_/DMSO post-treatment and LBL process, indicating that the surfaces of one-, two-, and three-layered H–PEDOT:PSS showed the same conformational structure.

**Figure 4:**
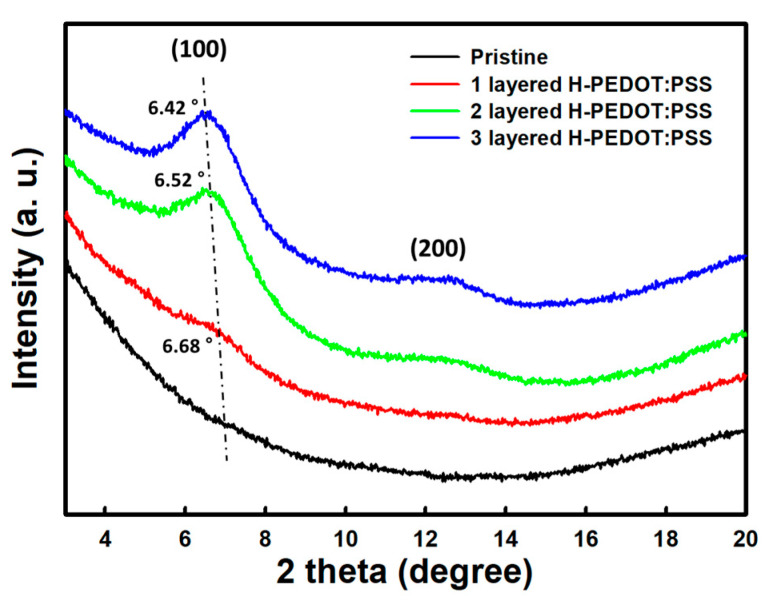
XRD patterns for the pristine PEDOT:PSS film and PEDOT:PSS films with the sulfuric acid etching treatment.

The crystallinities of the PEDOT:PSS thin films were analyzed by XRD, as shown in Figure 4. The PEDOT:PSS thin films were coated on a glass substrate and all samples were adjusted to the same 60-nm thickness. To obtain the distinct tendency, the samples were measured by in-plane diffraction technique. In the pristine PEDOT:PSS thin film, no obvious peak was observed because of the rich-PSS, located at the upper layer. This means that pristine PEDOT:PSS has an entirely amorphous structure. The lower two-theta angle under 18° are attributed to the lamellar stacking distance *d*_(100)_ of the distinct alternative molecular structure of PEDOT and PSS [21]. In other samples, the two diffraction peaks are attributed to the *d*_(100)_ between the successive PEDOT backbone chains. The peak at two-theta = 6.68° shifted to 6.42°, which suggests a broadened *d*_(100)_ spacing [22,23]. Its shift to a lower angle corresponds to the change from a coiled structure to a planar lamellar stacked structure. Moreover, the peak became sharper and more distinct with the repetitive H_2_SO_4_/DMSO post-treatment and LBL process, which induced ordered and crystalline molecular structures. This is because of the effect of the arrangement of the PEDOT chain through sulfuric acid and the removal of amorphous PSS [24].

**Figure 5:**
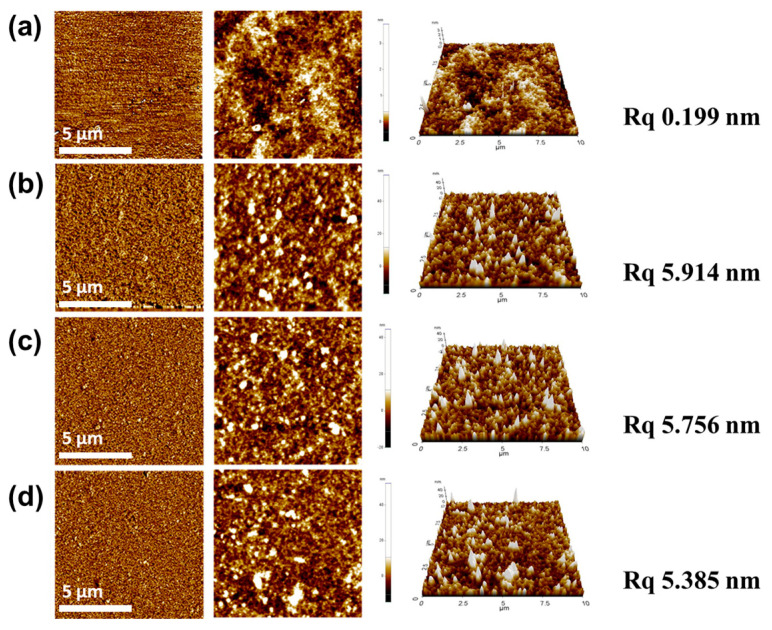
Atomic force microscopy (AFM) topographic (**left**), phase (**middle**), and 3D images (**right**) of (**a**) pristine PEDOT:PSS, (**b**) one-layered H-PEDOT:PSS, (**c**) two-layered H-PEDOT:PSS and (**d**) three-layered H-PEDOT:PSS thin films.

In the case of the morphology of the PEDOT:PSS thin films, the AFM images were measured, as shown in Figure 5. The pristine PEDOT:PSS showed an uncertain boundary between PEDOT and PSS, which presents amorphous structures. In contrast, the multi-layered PEDOT:PSS with the H_2_SO_4_/DMSO post-treatment and the LBL process showed a distinct phase separation of PEDOT and PSS grains. The PEDOT grains were aggregated using DMSO and H_2_SO_4_. The roughness of the H_2_SO_4_/DMSO post-treated PEDOT:PSS thin films was higher than that of the pristine thin film [25,26]. There was no difference in the tendency with the number of layers in PEDOT:PSS thin films.

**Figure 6:**
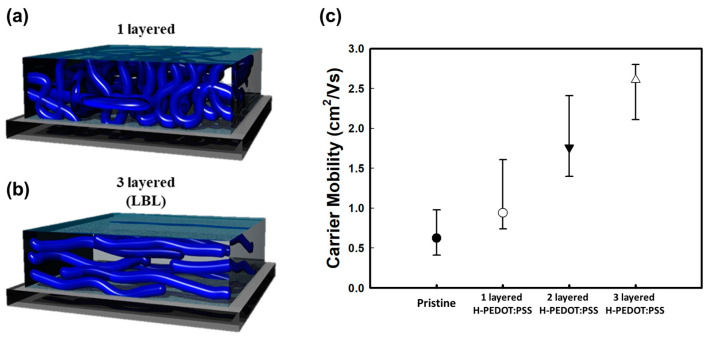
The post-treated H-PEDOT:PSS thin films with (**a**) one-layered and (**b**) three-layered structures. (**c**) Carrier mobility of PEDOT:PSS thin films at room temperature.

The improvement of electrical conductivity through the H_2_SO_4_/DMSO treatment and the LBL process was further studied by carrier mobility. The carrier mobility and concentration affect the electrical conductivity of PEDOT:PSS thin films [27]. The one-layered rich-PEDOT layer showed a relatively amorphous structure with respect to the multi-layered rich-PEDOT layers with linear structures, as shown in Figure 6a,b. The lamellar stacked PEDOT chains with linear structures enhanced the mobility of the hole transfer. The carrier mobility was gradually increased by a layer-by-layer process under the same thickness condition (Figure 6c). The highest electrical conductivity of PEDOT:PSS thin films with ordered structures through the H_2_SO_4_/DMSO post-treatment and LBL process was confirmed by enhancement of carrier mobility.

## 4. Conclusions

The two-dimensional lamellar stacked PEDOT:PSS thin film was prepared by a facile method consisting of etching and an LBL process. The coating conditions and post-treatment process, in order to fabricate the ordered PEDOT:PSS thin films, were optimized to a three-layered structure with a thickness of 60 nm. The relatively simple process induced the rearranged molecular structure of the PEDOT chain from a coiled to planar structure and dramatic improvement in the electrical properties, etching the rich-PSS layer using a sulfuric acid solution and repeatedly stacking the rich-PEDOT layer on it by LBL process. Through XRD analysis, it was found that the crystallinity of the lamellar structure of the PEDOT:PSS thin film is improved by etching and LBL processes, which implies a uniform electrical pathway. Thus, it can be considered as the main factor of the improvement of electrical conductivity. The carrier mobility increased from 0.62 to 2.80 cm^2^ V^−1^ s^−1^ through a repeated simple process, and it led to an increase in electrical conductivity from 801 to 3026 S cm^−1^ (3.8 times). This can be used as an organic electrode material for electronic devices such as organic thin film transistors or sensors.

## Figures and Tables

**Table 1:** The detailed sheet resistance, film thickness, and electrical conductivity data of PEDOT:PSS thin films according to H_2_SO_4_/DMSO post-treatment and LBL process.

Sample		Untreated	H	HS	HSH	HSHS	HSHSH
**Pristine** **PEDOT:PSS**	Sheet resistance (Ω sq^−1^)	204	-	-	-	-	-
	Film thickness (nm)	61	-	-	-	-	-
	Conductivity (S cm^−1^)	802	-	-	-	-	-
**1 layered** **H-PEDOT:PSS**	Sheet resistance (Ω sq^−1^)	75	75	-	-	-	-
	Film thickness (nm)	170	64	-	-	-	-
	Conductivity (S cm^−1^)	788	2091	-	-	-	-
**2 layered** **H-PEDOT:PSS**	Sheet resistance (Ω sq^−1^)	144	155	70	71	-	-
	Film thickness (nm)	88	30	90	58	-	-
	Conductivity (S cm^−1^)	792	2125	1591	2420	-	-
**3 layered** **H-PEDOT:PSS**	Sheet resistance (Ω sq^−1^)	172	175	72	79	50	53
	Film thickness (nm)	73	26	84	48	110	63
	Conductivity (S cm^−1^)	800	2199	1660	2645	1825	3026

Untreated: Single-layer coating process without post-treatment. H: H_2_SO_4_/DMSO post-treatment process. S: Layer-by-layer (LBL) coating process. H-PEDOT:PSS: Lamellar stacked PEDOT:PSS treated by H_2_SO_4_/DMSO and LBL process.
